# Two separate and important points that should not be forgotten among the consequences of the pandemic: psychology of healthcare workers and readmission of discharged COVID-19 patients

**DOI:** 10.3906/sag-2012-141

**Published:** 2021-06-28

**Authors:** Oğuz Abdullah UYAROĞLU

**Affiliations:** 1 Division of General Internal Medicine, Department of Internal Medicine, Faculty of Medicine, Hacettepe University, Ankara Turkey

## To the editor,

With great interest, I read the article by  ift iler et al. [1] about the COVID-19 scientific publications from Turkey. As stated in the article, humanity has been tackling with a dual threat; the pandemic and its socioeconomic consequences. However, apart from this dual threat, the greatest area of struggle is psychological. A public survey during the initial phase of the COVID-19 outbreak in China showed that 28.8% of the participants reported moderate-to-severe anxiety and 53.8% of the respondents rated the psychological impact of the outbreak as moderate or severe [2]. With the onset of the pandemic, increasing anxiety and panic in all communities has also seriously affected healthcare workers (HCWs), who have the critical role in the global response against COVID-19. Psychological status of HCWs during the pandemic is an important issue worth discussing. We see that you have overlooked this topic. Although there were 9 original articles [3–11] from Turkey indexed in LitCovid between the dates specified in your study, we have not seen any article about this topic among the “selected” articles. The study held in our hospital, namely “Evaluation of the effect of COVID-19 pandemic on anxiety severity of physicians working in the internal medicine department of a tertiary care hospital: a cross-sectional survey” yielded that particularly female doctors and physicians who have elderly family members and family members with chronic medical conditions showed more severe symptoms of anxiety [3]. Our study is one of the first studies published from Turkey to draw attention to the psychological consequences and risk factors for higher anxiety level of HCWs. Maintaining an adequate and physically healthy healthcare workforce in the still not waning pandemic requires an adequate number of mentally fit HCWs with abilities to care for high-volume patients under pressure.

The second subject I would like to draw your attention to is “readmissions”. Published data covers mostly information on the clinical features, laboratory findings, and treatment of patients hospitalized with the diagnosis of COVID-19; however, many of the COVID-19 patients are discharged with quarantine suggestions and treatment at home. There is limited data on whether these patients have fully recovered, presented again with persistent or new symptoms, or been readmitted. The readmission data of COVID-19 patients are still scarce. We examined the 30-day readmission rates of mild- and moderate-severity COVID-19 patients discharged from our tertiary care university hospital [12]. Prolonged fever and persistent cough were the most common complaints on readmission. The percentage of patients with accompanying hypertension and malignancy were higher among readmitted patients. To the best of our knowledge, this is one of the first studies exploring 30-day readmission rate of COVID-19 patients in the literature from a “quality of care” point of view and the first in Turkey. Since this article has been indexed in LitCovid very recently, it might have been missed in the article by  ift iler and colleagues; hence, it is not among the “selected” publications in your study.

We thank you for your great work on the review of the scientific contributions to LitCovid from Turkey. To contribute to this great work, we wanted to share this letter with you in order to attract the attention to two critical issues in COVID-19 areas: “psychological well-being of HCWs during the pandemic” and “readmission of discharged COVID-19 patients”.

**Key words:**Anxiety, healthcare workers, COVID-19, readmission

## Disclaimers

The author received no financial support and/or specific funding for this work.
